# Keeping vigil over the profession: a grounded theory of the context of nurse anaesthesia practice

**DOI:** 10.1186/1472-6955-9-13

**Published:** 2010-07-15

**Authors:** Rita S Schreiber, Marjorie A MacDonald

**Affiliations:** 1 School of Nursing University of Victoria, P.O. Box 1700, Victoria, V8W 2Y2, BC Canada

## Abstract

**Background:**

Nurse anaesthetists in the US have faced continued, repeated challenges to their profession. Regardless, they have met these challenges and have established themselves as major anaesthesia care providers. In this paper we address the research question: How do certified registered nurse anaesthetists (CRNAs) manage the socio-political context in which they provide care for their patients?

**Methods:**

Grounded theory was used to explore how nurse anaesthetists protect and promote their profession. Purposive, snowball, and theoretical sampling was used and data were collected through participant observation and interviews conducted at a conference of the professional association, an educational program, by telephone, email exchanges, and time spent in operating rooms and an outpatient surgical clinic. Analysis included coding at increasingly abstract levels and constant comparison.

**Results:**

The basic social process identified was Keeping Vigil Over the Profession, which explains how nurse anaesthetists protect and promote their profession. It is comprised of three contextual categories: Establishing Public Credibility through regulatory and educational standards, Political Vigilance and taking action in governmental and policy arenas, and Tending the Flock through a continuous information loop between local and administrative/political levels.

**Conclusions:**

From our study of the context of nurse anaesthesia practice, it is clear that CRNAs are dedicated to protecting their ability to provide high quality patient care by maintaining constant vigilance over their profession.

## Background

In 1931, Agatha Hodgins brought together in Cleveland 40 nurse-anaesthetists from 12 states to form the National Association of Nurse Anaesthetists (NANA--later American Association of Nurse Anesthetists--AANA). Within weeks, state organizations began forming, and almost immediately the profession faced its first legal challenge; in California Dagmar Nelson was charged with practicing medicine without a license. After a lengthy court battle, in 1934, the state Supreme Court ruled that nurse anaesthesia was not the illegal practice of medicine, and that anaesthesia performed by a nurse was nursing. This was only the first of many legal and political challenges faced by nurse anaesthetists, thus the need to Keep Vigil Over the Profession has always been present for them.

In an earlier report [1] we presented a grounded theory of nurse anaesthesia practice and its relationship to nursing and nursing knowledge: Keeping Vigil Over the Patient. In this paper we report findings related to the study question: How do certified registered nurse anaesthetists (CRNAs) manage the sociopolitical context in which they provide care for their patients?

## Methods

We used grounded theory to explore CRNA practice. As a qualitative, systematic approach, grounded theory (GT) is often used to explore social processes in context. GT involves the iterative collection and analysis of data to formulate explanatory theories grounded in the world of participants [2]. GT was originally developed by Glaser and Strauss [3] and refined by others, including Glaser [4], Strauss [5], Strauss and Corbin [6], Schreiber and Stern [7], and Charmaz [8]. Because we were interested in the processes that CRNAs use as they enact their role, GT was the method of choice. We were influenced by Strauss [5,9] in our examination of the contextual influences on situated interaction and CRNA practice.

Consistent with GT methodology, we sought variation in data sources and participant experiences, using multiple techniques. First, we attended the 2006 meeting of the American Association of Nurse Anesthetists (AANA), where we conducted participant observation, used purposive and snowball sampling to recruit participants, conducted interviews (n = 18), gathered documents, and visited and examined the association's archives. We interviewed key informants about socio-political matters like regulatory and legal issues, scope of practice, billing and reimbursement. Scientific sessions, convention rituals, and mentoring relationships were observed. We also conducted e-mail and telephone follow-up to clarify issues. Theoretical sampling led us to conduct additional interviews with new informants. Most interviews were taped and transcribed verbatim; interviews not recorded were documented in field notes.

Participants' years of CRNA experience ranged from student through 40 years, and participants represented a minimum of 10 states; many had military experience, had worked in several states in both rural and urban settings, and some had worked in *locum tenens *positions. Eighteen of 41 participants were men.

Based on emerging theoretical concepts, we conducted a site visit to a small city in the American Northwest to explore the context of CRNA practice. We observed anaesthesia practice in an outpatient clinic and in two hospitals, visited an educational program and observed practicing CRNAs supervising students. We gathered data through document review, formal and informal interviews, and observations in clinical settings. Throughout the study we wrote or recorded field notes documenting our observations, interactions, and analytic ideas.

### Data Analysis

Analysis began with entry to the field and continued concurrently with data collection. Throughout we met and discussed emerging findings and agreed on interpretations and memoing. We assigned increasingly abstract codes and compared incoming data to other data, to concepts, and to emerging categories. We constantly made comparisons among ideas about actions and interactions occurring in the data. Finally, we constructed a theory of nurse anaesthesia practice, **Keeping Vigil **comprising two major categories. Keeping Vigil Over the Patient, which theorizes nurse anaesthesia practice and is reported elsewhere [1] takes place within a larger sociopolitical context, Keeping Vigil Over the Profession, presented in this paper.

### Rigour

We used widely accepted techniques to ensure rigour, including constant comparison, "negative" case analysis, participant checks, persistent observation, and prolonged engagement in the field [3,10]. We also used peer debriefing within a GT seminar [11]. We listened to the tapes repeatedly, while reading and re-reading the text. Selected participants reviewed the findings and provided feedback to ensure our interpretations were consistent with their experience and understanding.

### Limitations

As in all qualitative research, findings cannot be generalized widely. However, if the findings fit in explaining other situations and circumstances, then they can be said to be theoretically generalizable [12,13]. Participants included only active members and employees of the AANA and it is unclear whether or how well these findings would explain the actions and interactions of non-active members or non-members. Yet, the AANA works to keep members actively informed and involved, and its membership includes an estimated 95% of nurse anesthetists in the country.

### Ethical Considerations

The study was conducted in accordance with the Canadian Tri-Council [14] guidelines on human subject research, including informed consent. The study received approval of the Human Research Ethics Committee of the University of Victoria and the Board of Directors of AANA.

## Results

**Figure 1:**
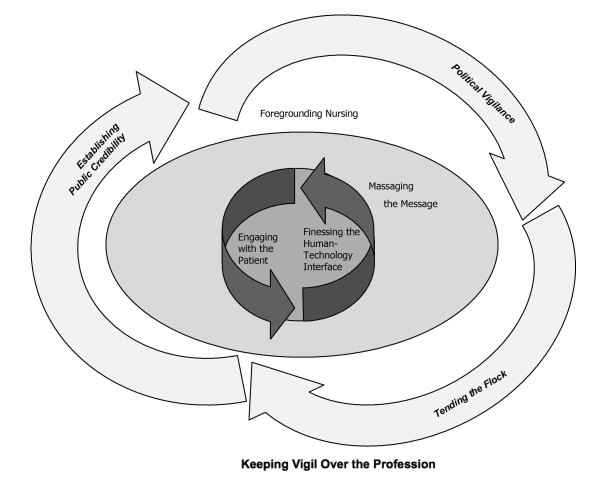
**Keeping Vigil Over the Profession**.

In this paper we report on Keeping Vigil Over the Profession comprising three major contextual categories that influence how CRNAs Keep Vigil Over the Patient: Establishing Public Credibility, Political Vigilance, and Tending the Flock. To keep vigil over the patient, CRNAs must also keep vigil over the profession (see Figure 1). This vigilance operates in the background of CRNA practice and works to buffer it from ongoing threats. Participants are convinced that without such vigilance, the continuous political and legal challenges launched against them would whittle away at CRNA practice, imposing ever-increasing limits on their scope. This vigilance also operates internally within the professional organization, working to keep the membership informed, cohesive, and speaking with a unified voice.

### Establishing Public Credibility

A primary concern of Hodgins and her colleagues that continues today is the need to establish the credibility of the nurse anaesthesia role in the eyes of the public. CRNAs must not only practice safely, but they must be seen to be practicing safely. Thus, nurse anaesthetists needed to set a high standard and demonstrate the safety and worth of their practice. In practice, they also needed to establish themselves as trustworthy, safe providers of anaesthesia care. They did this by adopting a protected title for nurse anaesthetists, establishing standards of practice and a standardized curriculum, as well as personal-professional credibility.

#### Adopting a title

Early in its history (1956), AANA members adopted the title Certified Registered Nurse Anaesthetist, or CRNA. This title recognizes the national certification that was already established and enshrined the "nurse" part of nurse anaesthesia, which was and continues to be viewed by practitioners as the foundation and spiritual home of their practice. The following quote is a typical example of the commitment to nursing within CRNA practice and identity:

I think the reason why nurse anaesthesia is a nursing role is that we are able to combine all that good science stuff along with our nursing stuff. You have an opportunity to take care of someone who is in a huge anxiety part of their life and alleviate that anxiety through just touching them on the shoulder, touching them on the head, telling them "This will be OK, and I'm going to take really good care of you." And to me, that's nursing.

So strong is the commitment to nursing that an attempt at an AANA meeting to drop "nurse" from the title was widely defeated, which is notable in light of the fact that, although affiliated with universities, approximately 40% of CNRA educational programs are still housed outside schools or departments of nursing.

Identifying an agreed-upon title for the role allowed the professional association to mount public relations campaigns about the role and the title, and enabled them to create and distribute recruitment materials to attract students to the profession. By having a professional name that was recognized and accepted by all, the group prevented many of the nomenclature arguments and irregularities within nursing that have dogged the more recent development of the nurse practitioner (NP) role in Canada where there is no inter-provincial agreement on title and title protection NPs.

#### Developing practice standards

Similarly, formation of NANA/AANA created a forum and the means to develop practice standards and establish curricula. Creation and ongoing revision of standards of practice promoted a high quality of care and helped ensure the status of the CRNA role. It further allowed members to define for the public the nature of their care, as well as identify a level of excellence for CRNAs to achieve. CRNAs could enumerate their qualifications to those who might question their preparation to provide safe care.

In addition, NANA/AANA approved educational programs, and laid the groundwork for the accreditation and certification processes that continue today, although AANA no longer accredits nurse anaesthesia programs or certifies CRNAs. In 1975, as a result of challenges by the American Society of Anaesthesiology (ASA) to the U.S. Office of Education, the AANA relinquished these responsibilities to five independent Councils. Since 1975, the AANA has been the sole professional organization that represents all CRNAs in all situations public and private at both state and federal legislative levels. CRNAs could now point to these developments to demonstrate the rigour of their training and their high practice standards. This works today both at the individual level, in establishing personal-professional credibility with physicians, and within institutions and the nursing community. It also works at the state and national level, in lobbying for legislation and regulation that support a broad scope of CRNA practice.

#### Establishing educational programs

The creation and adoption of a standardized curriculum for preparation of nurse anaesthetists began in 1935, with a report from the AANA Education Committee. The report contained recommendations regarding subjects to be taught, hours of classroom and clinical instruction, and a minimum number of cases to be administered by the student to get a diploma. The AANA report served the same purpose that the 1910 Flexner Report did for physicians, using educational standards to position CRNAs and their practice in a politically advantageous place. To support members in meeting these educational requirements, AANA sponsored refresher courses between 1947 and 1951.

Over time, CRNA educational preparation has been increased. This began with requirements for specific program lengths, which kept expanding; however, it took a long time for CRNAs to agree on the need for baccalaureate or graduate education. For example, the "1985 Proposal" to mandate a minimum of baccalaureate preparation, failed at the AANA conference that year. Nonetheless, there was a general recognition of a need for degree level preparation, and in 1998 it became a requirement that CRNAs be prepared at the graduate (master's) level. Presently discussions are underway regarding doctoral preparation, in light of the American Association of Colleges of Nursing (AACN) plan to advance preparation for NPs (and CRNAs) to a clinical doctorate. There is ambivalence among CRNAs with respect to this proposal because some are concerned that the DNP (doctorate in nursing practice) appears to be more relevant to NPs and is not specific to CRNA needs. The debate is ongoing [15,16] although participants recognize the inevitability of a requirement for doctoral preparation and the importance of moving in parallel with other advanced practice nurses (APNs). Nonetheless, the AANA has issued a position statement that all CRNAs graduating in 2025 and beyond should be accredited at the doctoral level.

The idea of a DNP and other clinical doctorates arose from recognition of the importance of practice-based higher education in providing safe patient care. Admittedly, some would argue that the promotion of clinical doctorates for APNs is, in part, a political maneuver "which creates parity with other healthcare professionals" [15]. Yet, this reinforces the ongoing challenges experienced by CRNAs and their need for constant political vigilance.

#### Establishing personal-professional credibility

In tandem with the AANA's activities to establish and enhance the credibility of the profession, individual practitioners have credibility issues as well. In addressing their own personal-professional credibility, CRNAs reinforce the collective work to establish the credibility of the profession in the public eye. Many participants talked about the need to establish their own personal-professional credibility in their relationships with physicians, and the documentary record shows that this has long been the case [16,17]. The earliest CRNAs worked closely over long periods of time with a single physician who fully supported their development as nurse anaesthetists. Examples of this include Agatha Hodgins and Dr. George Crile at the Lakeside Hospital in Cleveland, or Alice Magaw and the Mayo brothers.

Participants described how they had to establish credibility when they encounter anaesthesiologists who are unfamiliar with, or resistant to, their practice or with CRNA practice in general. For these anaesthesiologists, seeing is believing, and credibility could only be achieved by the CRNA demonstrating competence and establishing a level of trust with medical staff, as described by this participant:

It takes time for them to have a level of trust. I think it all comes down to trust. And I don't think I could go into any practice and work with an anaesthesiologist without earning their respect and trust, and then it becomes more of a supportive type role.... What they say behind our backs? Probably what we say behind their backs, you know? There's good and there's bad and um, but I think it just comes down to earning respect and trust and I think it goes both ways.

This participant hints at the intricate relationship between CRNAs and anaesthesiologists, in that they intentionally focus on the well being of the patient and providing high quality care, which diffuses conflict in the operating room [18].

##### Maintaining Sociopolitical Vigilance

From the beginning, participants identified that CRNAs have faced ongoing challenges requiring constant vigilance of the socio-political context in which anaesthesia practice takes place. Maintaining socio-political vigilance includes two subcategories: monitoring challenges and politicking.

#### Monitoring challenges

The AANA and its members continuously scan the environment for threats to the profession. Our analysis of participants' descriptions revealed three related types of threats faced by CRNAs: challenges to professional autonomy, challenges to the scope of practice, and encroachment on their practice by others, notably the anaesthesia assistant (AA). Many threats have come from the ASA or their proxies, resulting in continuous lobbying and counter-lobbying by AANA and its members at the state and federal level over regulations and legislation related to CNRA autonomy, scope of practice, and reimbursement for services.

CRNA autonomy has been contested periodically in American courts, as physicians have repeatedly sued (largely unsuccessfully) CRNAs for practicing medicine without a license. Although there is growing recognition at the regulatory level that the nature of practice overlaps between and among health professions, there are ongoing attempts by anaesthesiologists to curtail autonomous CRNA practice by lobbying for supervisory requirements for state Medicare/Medicaid and third-party payers (private insurance companies). One participant spoke of the political landscape in her state as, "They [state medical association] have 20 lobbyists to our one. Big machine." Challenging autonomous practice via payment schemes is a common strategy to reach the same outcome through the back door, and such challenges are frequent. As one participant described this, "The only downside [to anaesthesia practice] is that it's so contentious. We have to fight for it all the time."

CRNAs also face challenges to their autonomy from hospital policies that require medical supervision of CRNA practice, regardless of the fact that there is no legislation requiring them to work under medical direction or supervision of an anaesthesiologist [19,20]. In teaching hospitals, the common practice is to have an anaesthesiologist "supervising" CRNAs and anaesthesia residents in two to four operating rooms. Often the anaesthesiologist's focus is on the practice of the residents, because the CRNAs are usually much more experienced and able to function by using the anaesthesiologist as a consultant, if at all. At the same time, this hierarchical arrangement appears as an optical illusion that somehow the anaesthesiologist is the "captain of the ship".

Related closely to threats to autonomy are local and national political struggles to safeguard the scope of practice and compensation [17]. Although the overall trend is toward fewer regulatory constraints to APN in the US [20], the medical establishment has successfully constrained the CRNA scope of practice in several states. For example, in some states CRNAs are unable to provide regional anaesthesia because of wording of the Medicare/Medicaid payment conditions. Interventional pain management by CRNAs, as might be provided in a pain clinic, is controversial, and in a state where medical organizations claim that it is solely the practice of medicine, CRNAs currently are restrained from performing certain pain management techniques. One participant explained:

The limits on CRNAs in Louisiana ... do not relate to Medicare/Medicaid billing. Rather, this was an outcome that emerged from dueling Board of Nursing and Board of Medicine positions and litigation that, unfortunately, did not end positively for the nurse anaesthetists ... it's clearly an aberration - at least to date - as no other state has yet adopted such a prohibition concerning nurse anaesthetists.

There are also states in which Medicare/Medicaid and/or third-party payers do not reimburse CRNAs at the same rate as anaesthesiologists performing the same service, and often there is no legal ban in place to forbid this practice.

At the same time CRNAs are also threatened by the encroachment of the anaesthesiologist assistant (AA) role. The AA role varies across the country, from that of the technician ensuring equipment and supplies are prepared for the day's surgeries to a "physician assistant specialist" [21], administering and monitoring anaesthesia under the direction of an anaesthesiologist. Currently, the AA role is regulated in a two states, although it exists under medical direction within hospitals, so it is difficult to estimate the number of recognized AAs practicing.

As can be seen, CRNAs face continuous threats to their profession. Both individually and collectively, they understand that their practice is contingent upon being able to identify, prevent, and address challenges to the profession.

#### Politicking

CRNAs recognize that they need to be effective in pushing back against possible impediments to the profession and the role. Nonetheless, as noted elsewhere [1], although the ASA and AANA are locked in conflict, CRNAs ensure that this conflict does not affect patient care. Given the nature and constancy of these threats, it has been necessary for CRNAs to be politically vigilant, acting jointly through the AANA, which works to keep its membership together and speaking with one voice. As a collective, AANA represents a critical mass of CRNAs, and has resources to provide its membership with the skills and key messages to be effective in promoting their profession. For example, the AANA has a branch office in Washington, DC, steps from the Capitol Building, where a full time professional lobbyist and up to six other staff monitor political happenings at the national and state levels. They prepare briefs and send out electronic updates and bulletins to the membership about matters such as progress of legislation, lobbying efforts and messages, and upcoming committee hearings. They also maintain a system to gather information about state and local initiatives with potential impact on practice, and to provide support and information to members to deal with these. Thus, mechanisms are in place and well practiced to launch public relations campaigns and counter-offensives as needed, which has enabled the group to speak with one voice, something they have continued to do over the years. To date, this has proven effective.

In addition to monitoring political developments, staff at the Washington, DC office also conducts an annual Assembly of States, a workshop aimed at providing participants with the skills needed to work effectively with the media and get their message across to the public in a clear manner. The workshop includes up to 10 participants per state, including state leadership and boards of directors. Over the course of a week, they learn about government, legislative initiatives, Medicare/Medicaid rules, the political process, and how to be useful to elected officials. The week culminates with groups of two to three participants going to the capital and meeting with their congressperson or senator, taking their key messages and practicing their new skills. Participants are then prepared to go back to their home states and become involved with promoting the profession at a political level. One CRNA noted the importance of this work:

That's one thing that's wonderful about the AANA. They try very hard to teach their people the political landscape and how to stay involved, because if you're not involved politically, others will legislate you out of business.

Thus, in these ways, CRNAs are able to protect their profession by Maintaining Political Vigilance. They are able to identify threats to their practice and work in a skilful way as individuals and as a collective to disarm challenges and promote the profession. With an estimated 65% of all anaesthesia given in the US administered by CRNAs, the results of their efforts are clear.

##### Tending the Flock

We were struck by the many ways in which CRNAs, individually and through AANA, attend to themselves and the collective membership. This is an empowered group of nurses, and the AANA conference was a stark contrast to the many other nursing conferences we have attended in Canada and the US. Because AANA represents virtually all CRNAs, and because threats to the profession are continuous, considerable effort is made to nurture and strengthen the group. This is done through two sub-processes, nurturing ourselves and celebrating traditions.

#### Nurturing ourselves

CRNAs nurture themselves by attending to their own health and the health and future of the profession. The health of AANA membership was a particular concern, and this was evident at the conference with early morning golf and walks, and a lifestyle booth with nutritional and other handouts, including a video on prevention and treatment of substance use. There was also a presentation on the high (10%) risk of substance use within anaesthesia practice (including both nurses and physicians), in which the presenter raised the issue of mandatory drug testing. One reason for the focus on substance use was the recent overdose death of a highly respected, much loved AANA president. This was not believed to be a planned suicide, but the result of an accidental overdose while self medicating. There was a general recognition that perfectionism and CRNAs' considerable knowledge of drugs could easily lead them to feel somewhat grandiose about their ability to handle drugs, as this participant explained:

So I can completely understand people saying, I know this drug, it can't happen to me, I know ... but um, it's very scary. And it's very sad and devastating and um, I just don't ever want to see any of my colleagues...and I know I will some day. I mean most of the senior people that I know, they all know people that it's happened to. So, I'm really hoping that can, the wellness initiative will really help our profession become stronger physically, mentally, spiritually, everything.

The forthrightness with which participants, both at the conference and during our site visit, talked about substance use was striking. We heard variations on this discussion throughout the study from many participants, some of whom were friends of the AANA president who died from an overdose.

CRNAs also nurture themselves through mentoring future generations of anaesthetists. Guiding students is a serious concern of CRNAs, and they talked with particular pride about this:

It's frequently mentioned that nurses eat their young and they do. They do. But nurse anaesthesia doesn't do that. We really... I don't think that you'll find anybody that says that we do. We really mentor because we have a nurse that we're mentoring to do what we do and that's different I think.

Participants were insistent that nurse anaesthesia instruction was qualitatively different than what medical residents receive, as these quotes demonstrate:

The difference between CRNA training and resident training is unbelievable, in terms of the mentoring and the attention. A resident gets...( ) ...give them a month one on one, month of July, one on one, whether it be with a senior resident or one of the CRNAs, one on one, and then you're [medical resident] out [on your own].

In fact, in my program, the residents told us that they felt we got better anaesthetic education, than they did because they were left to their own devices to educate themselves, whereas we were given formalized instruction and testing.

Participants emphasized that their tradition of mentoring students and monitoring their learning closely helped to prevent mistakes. In fact, CRNAs' malpractice costs have decreased by 42% in the past 18 years [16] and malpractice rates for CRNAs are reported to be lower than those of anaesthesiologists [22].

We observed a CRNA working with a student in difficulty, and saw her posing questions to help him focus on what was salient, asking, for example, "Given this patient's age and weight, where do you want to keep his BP?" In this way, she was asking the student to bring classroom learning into the practice arena while keeping an eye on the parameters she was willing to allow him.

Nurturing ourselves was also evident at the conference, where there were opportunities for CRNAs to sponsor students by paying their registration fees and bringing them along as shadows throughout the conference, introducing them to others and explaining what they were seeing. All CRNA programs require students to become associate members of the AANA, which allows them to become non-voting members of national and state committees, and further mentors them to become involved in the AANA when they graduate. Some programs pay students' AANA dues while they are in the program. During their programs, students are required to attend and participate in either national or regional/state meetings of AANA, and report back to their student colleagues. Students are encouraged to present posters at conferences, usually on their final research projects, and we observed many excellent examples. In addition, CRNAs are expected to be preceptors to students as part of their daily work and take this on willingly. In these ways, the fostering and guidance of students was a serious responsibility, and a part of Tending the Flock on which CRNAs took pride in themselves.

#### Celebrating traditions

CRNAs also Tended the Flock through the many traditions they celebrated. This was particularly evident at the 75^th ^anniversary meeting of AANA's founding, where there was a Pioneer's Breakfast, a meeting of the history committee, and where the organization's archives were displayed. Each of these communicated the diverse and important CRNA traditions to the flock. There was considerable formality at the opening and closing ceremonies, much more than we were used to at Canadian events. For example, at these events, the officers and board members, formally dressed, were introduced to applause as they marched down a spotlighted aisle to the front podium. The happenings were all projected on giant screens. At the banquet, the persons accompanying these dignitaries (partners or family members of whatever gender or sex) were also introduced in this way. The events were thoroughly choreographed preventing awkward moments as people received various awards, shook hands, turned to be photographed, and returned to their places, all set to a trumpet fanfare. Those members in the military attended in full dress uniform, presenting a striking vision.

At the banquet, members were seated by state and as each state was called (with appropriate music and film footage on the big screens), members jumped up, waved their napkins, and loudly cheered themselves and their state(s). Some went to considerable effort to produce costumes to reinforce their various allegiances. It resembled a pep rally, with a roll call of states, territories, public health, and the military that took about an hour as a prelude to dinner. Throughout these events, there was a strong undercurrent of patriotism. In this way, there was serious intent along with an element of fun as these events unfolded, and these traditions fostered and strengthened bonds within the membership.

## Discussion

In this report, we theorized the multi-level context of nurse anaesthesia practice in the US to provide an understanding of the significant contextual influences on practice. In a related article [1], we presented a substantive theory of CRNA practice, Keeping Vigil over the Patient. As qualitative researchers, we acknowledge these findings represent our interpretation of the data from the perspective of "outsiders"-- nurses who are not nurse anaesthetists. This may preclude a full understanding of CRNA practice and the influences on that practice as experienced by CRNAs. We believe, however, that we offer a useful perspective on one area of APN that has relevance to the broader profession. There is much we can learn from the way CRNAs in the US ensure safe and high quality care for their patients, manage the challenges to their professional autonomy, and promote and advance the wellbeing of their members.

What stood out for us were two of the three categories in Keeping Vigil over the Profession: *Political Vigilance *and *Tending the Flock*. The first category, *Establishing Public Credibility *was not new to us, nor is it new to APN. The processes of adopting a title, developing practice standards, developing regulatory and credentialing mechanisms, and establishing accredited educational programs are consistent with developments in the larger APN arena [23]. What is perhaps most noteworthy is that nurse anaesthesia was the first specialty group in nursing to do this [16]. Much of this development was related to challenges in establishing credibility with the medical profession and the public at large, but also with nursing colleagues. The relationship between CRNAs and the larger profession of nursing, primarily as represented by their professional associations (AANA and ANA), has not always been an easy one and tensions remain. There is, however, growing professional collaboration between these groups. For example, there is an increasing trend to house nurse anaesthesia programs in colleges or schools of nursing [16]. In the early years, when anaesthesia programs were introduced, there was often opposition to having these programs in university nursing schools.

The other observation we made is the explicit link between establishing personal-professional credibility and collective credibility for the profession at large. The personal really is political. Early CRNAs worked closely with pioneer surgeons to provide anaesthesia in ground-breaking cases, thereby giving them credibility to develop practice and educational standards [1,16,17]. CRNAs today, although not working solely with specific physicians, establish trust and credibility with surgeons and anaesthesiologists in practice, which confirms collective credibility. It is possible that the credibility of CRNAs and their record of safe and competent practice fuels opposition to the CRNA role by the ASA. This relationship between personal credibility and collective professional credibility has been noted in relation to the acceptance of other APN roles, particularly nurse practitioners [24,25].

The category of *Political Vigilance *was one of the findings that most impressed us, although this type of vigilance is not unique to CRNAs or the AANA. What is unique is the widespread acceptance of, and engagement in, political activities among CRNAs. In fact, political activism is a dominant value among nurse anaesthetists and CRNAs engage in political activities because it is among the "dominant rules of behaviour" [25]. What is important about this political activity is that it is not just about vigilance in defending the practice rights of the profession, but vigilance in patient care and safety. We have not seen the value for political activism reported elsewhere as being so explicitly a core value or as prevalent in practice among other nursing specialty or professional groups. The importance of political activism to nurse anaesthetists is also implicitly reflected in the Nurse Anaesthesia Practice Model [26], in which one of the essential professional values is a social one. Value is placed on enhancing and preserving the CRNA role in the health care system in society at large, on the legal responsibility for competent care, and on continuous improvement in standards of care, education, and collaboration.

The importance of political vigilance is evident in the successful countering of many court challenges brought against CRNAs over the years for practicing medicine without a license. We know that other advanced practice nurses have faced similar challenges but it seems that the frequency and intensity of challenges has been greater for CRNAs, particularly in the last two decades [27]. It is not just the legal challenges that require maintaining vigilance, but also the attacks against scope of practice at the policy and institutional level as well. In fact, our CRNA participants told us that the constraints that physicians attempt to impose on their practice are primarily at the policy level, but may relate to institutional, state or national policies.

We wonder if the intensity of the legal and policy constraints that anaesthesiology groups attempt to impose on CRNA practice relate to the extent of competition they perceive. Nurse practitioners often practice in settings and with populations that do not always place them in direct competition with physicians. The same is true for CRNAs working in rural settings, where they are the primary anaesthesia providers. However, when CRNAs work side by side with anaesthesiologists in cities, their scope of practice overlaps almost entirely. Because of this overlap, there exists the potential for CRNAs to replace anaesthesiologists. This has led to anaesthesiologists lobbying for the "team model" of practice in which an anaesthesiologist "supervises" CRNAs. Although supervision may be a myth at the practice level [28], it has been successful in sustaining physician incomes in the face of any competition. Despite the effort it takes the AANA to counter these policy constraints, the fact that they are not enshrined in legislation provides opportunities to lobby to support and advance CRNA practice. But, constant vigilance is needed.

The third category, *Tending the Flock*, was perhaps the most surprising and exciting finding in our study. We observed CRNAs to be an empowered group of nurses and their practices in nurturing themselves stood in stark contrast to our own experiences in nursing, and to our observations at professional meetings and conferences. Our participants talked extensively about working to mentor the next generation of practitioners, and their willingness to engage in this mentoring role. Our own experience has been that it is challenging to find practice opportunities for students and we often face unwillingness among some practitioners to take on a mentoring role, often because of challenges in the workplace. This did not appear to be the case among the CRNAs in this study.

The frequent comments by participants that CRNAs do not "eat their young" was particularly telling. We could not find anything in the literature suggesting that the kind of horizontal violence or bullying characterized by the ugly metaphor of "eating our young" was evident in CRNA practice. We found several recent articles about this phenomenon in perioperative nursing practice [29,30] and other areas of nursing [31-33]. The usual explanation for such behaviour lies in theories of oppression and feminism, and in particular, the theory of oppressed group behaviour, popularized in nursing by Roberts [34] and taken up by other authors [35-37]. Because nursing is largely a female profession, it is vulnerable to sex-role stereotyping and gender oppression [38]. Although Farrell [31,39] has challenged the reliance on theories of oppression as the primary explanation for horizontal violence, he and others provide evidence that intra-professional conflict and horizontal violence is common in nursing. That we did not find it in our study suggests that there are unique characteristics of CRNAs, their role, and the organizational arrangements within which they work that make this type of behaviour unlikely. We also explored the literature for evidence of horizontal oppression among other advanced practice roles and found that it was also limited [24]. The high degree of professional autonomy, the higher level of education, and the broad scope of responsibility and accountability of APNs may account for this. In addition, the fact that 46% of CRNAs in the US are men [40] may have changed intraprofessional dynamics such that the largely gendered aspect of horizontal violence in other areas of nursing is ameliorated in CRNA practice.

## Conclusions

From our study of the context of nurse anaesthesia practice, it is clear that CRNAs are dedicated to protecting their ability to provide high quality patient care by maintaining constant vigilance over their profession. To do this, they actively engage in establishing their own personal credibility, as well as the credibility of the profession. They have a strong commitment to nurturing themselves and the next generation of CRNAs through *Tending the Flock*, which ensures strong and confident practitioners and a powerful professional association. Their commitment to political vigilance not only protects patients, but also ensures a place for CRNAs in the US health care system.

## Competing interests

The authors declare that they have no competing interests.

## Authors' contributions

Both authors participated in the conceptualization and design of the study and collected data in all its phases. RS conducted telephone interview and email exchanges to saturate the data set. Both authors participated in data analysis. RS prepared the first draft of the results section and MM prepared the first draft of the discussion. Both authors revised, edited, read and approved the final manuscript.

## Pre-publication history

The pre-publication history for this paper can be accessed here:

http://www.biomedcentral.com/1472-6955/9/13/prepub
